# Supplementary material for: Hall Effect Anisotropy in the Paramagnetic Phase of Ho0.8Lu0.2B12 Induced by Dynamic Charge Stripes
Source: Molecules. 2023 Jan 9;28(2):676. doi: 10.3390/molecules28020676 (PMC9863903; doi:10.3390/molecules28020676)
Supplement: Supplementary file 1 [file molecules-28-00676-s001.zip › molecules-2050624-supplementary.pdf]

Supplementary Materials to the article

Hall effect anisotropy in the paramagnetic phase of $Ho_{0.8}Lu_{0.2}B_{12}$ induced by dynamic charge stripes

A.L. Khoroshilov[a*], K.M.Krasikov[a], A.N.Azarevich[a,b], A.V. Bogach[a], V.V. Glushkov[a], V.N. Krasnorussky[a,c], V.V. Voronov[a], N.Yu. Shitsevalova[d], V.B. Filipov[d], S. Gabáni[e], K. Flachbart[e], N. E. Sluchanko[a]

[a]*Prokhorov General Physics Institute of the Russian Academy of Sciences, Vavilova 38, 119991 Moscow, Russia*

[b]*Moscow Institute of Physics and Technology (State University), Moscow Region 141700 Russia*

[c]*Vereshchagin Institute for High Pressure Physics of RAS, 14 Kaluzhskoe Shosse, 142190 Troitsk, Russia*

[d]*Institute for Problems of Materials Science, NASU, Krzhizhanovsky str., 3, 03142 Kyiv, Ukraine*

[e]*Institute of Experimental Physics SAS, Watsonova 47, 04001 Košice, Slovakia*

**E-mail: nes@lt.gpi.ru*

1. Relationship between (a) the conventional field-sweep technique for Hall effect measurements and (b) the scheme with step-by-step sample rotation in external magnetic field.

Hall resistivity of the samples was detected in two ways (see Fig. S1a and Fig. S1b below, correspondingly):

(a) The conventional field-sweep technique for Hall effect measurements, in which the value of Hall resistivity was calculated as $\rho_H/H = [(V_H(+H) – V_H(–H))/(2I)]\cdot d/H$, where I is the measuring current through the sample, d is the thickness of the sample, and $V_H(+/–H)$ are the voltages measured from Hall probes in two opposite directions of the external magnetic field **n**//**H**$\perp$**I** (**n** is normal to the lateral surface of sample).

(b) The angular dependences of Hall resistivity were obtained using a measuring cell of original design, which provides the rotation of vector **H** located in the plane (1-10) perpendicular to the current direction **I** || [1-10] with a minimum step $\Delta\varphi = 0.4°$ (see the schematic view in the inset of Fig. 2a in the manuscript). Measurements were carried out in a wide temperature range 1.9 - 300 K in magnetic fields up to 80 kOe, the angle φ = **n**^**H** between the direction of the normal vector **n** and external magnetic field **H** varied in the range φ = 0 - 360°. The setup for measurements was equipped with a stepper motor which enabled automatic control of the stepwise sample rotation.

High accuracy of temperature stabilization ($\Delta T \approx 0.002$ K in the range 1.9 – 7 K) and magnetic field stability ($\Delta H \approx 2$ Oe) was ensured, respectively, by Cryotel TC 1.5/300 temperature controller and Cryotel SMPS 100 superconducting magnet power supply in combination with a CERNOX 1050 thermometer and n-InSb Hall sensors.

Thus, panel (a) in Fig. S1 below shows the magnetic field dependencies of the reduced Hall resistivity $-\rho_H/H(H)$ at T = 2.1 K for H ≤ 80 kOe. Panel (b) in Fig. S1 shows the angular dependences of reduced Hall resistivity $\rho_H/H(\varphi)$ in magnetic field H = 80 kOe at T = 2.1 K. The dotted lines indicate the correspondence between the data at H = 80 kOe on field-sweep $-\rho_H/H(H)$ curves at $\varphi = 0°$ and 180°, and the angular dependences $\rho_H/H(\varphi)$ recorded in the range $\varphi = 0° - 360°$. For convenience, in Fig. S1b, the curves for all samples with different **n** except **n**||[110] are shifted by appropriate angles to match the respective normal directions. The location of these normal vectors **n**||**H** is marked with arrows in Fig. S1b, the upper axis legend indicates the crystallographic directions.

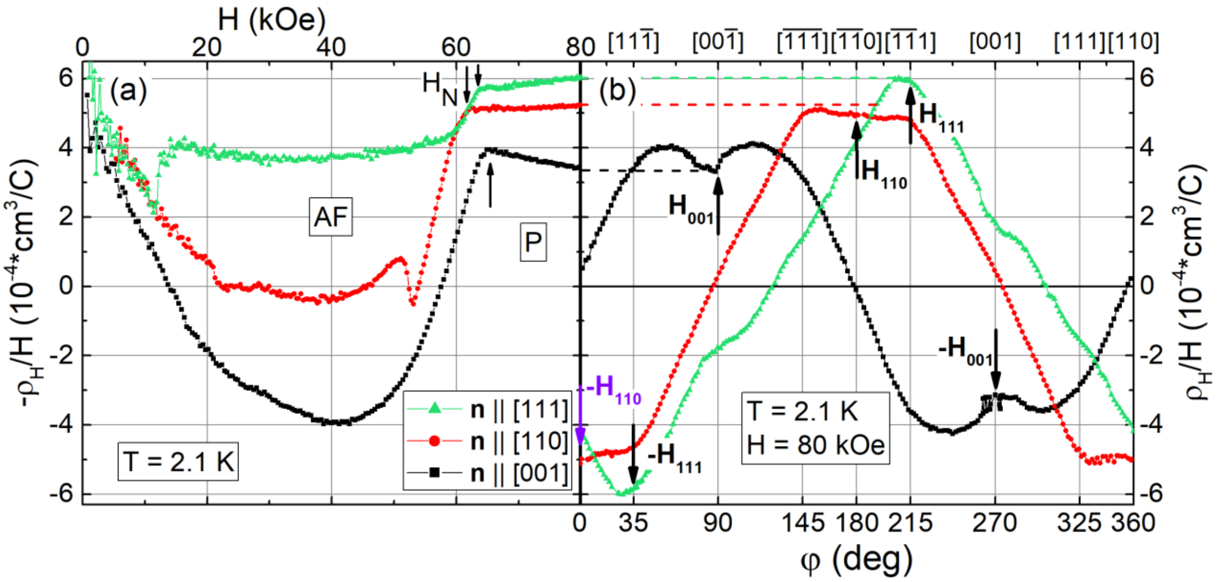

Figure S1. Relationship between (a) the conventional field-sweep scheme of Hall effect measurements with two opposite orientations of magnetic field strength ±**H**||**n** and (b) the scheme with step-by-step sample rotation in the plane (1-10) perpendicular to the measuring current **I** || [1-10] (see also inset in Fig. 2a of the paper).

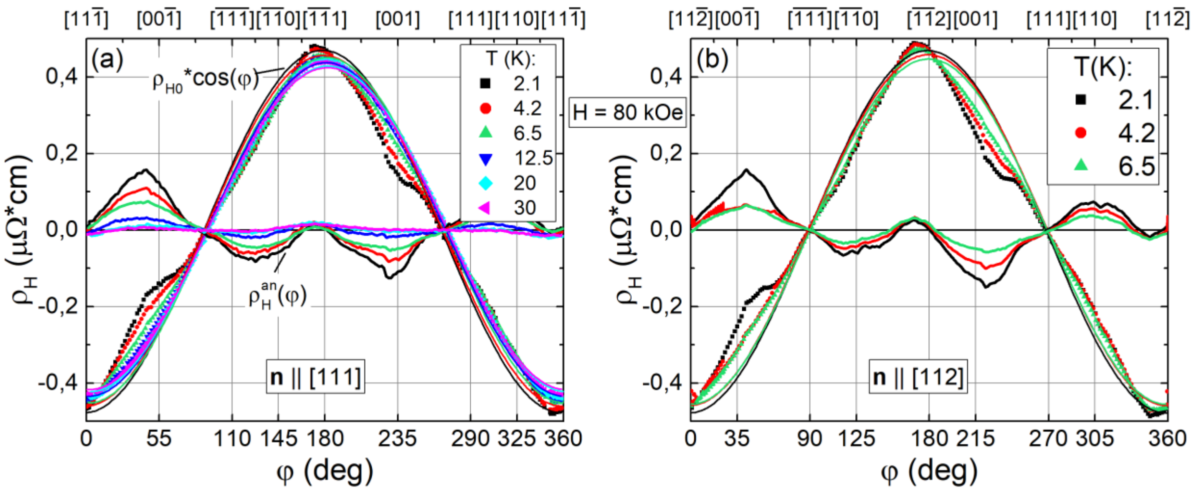

Figure S2. (a) - (b) Approximation by Eq. (2) in the article of the angular dependences of Hall resistivity $\rho_H(\varphi)$ for H = 80 kOe in the temperature range 2.1 - 30 K for **n**||[111] and **n**|| [112]. Symbols show the experimental values of $\rho_H(\varphi)$, thin curves present the isotropic contribution $f_{cos}(\varphi) \approx \rho_{H0} \cdot \cos(\varphi)$, thick curves demonstrate the anisotropic contribution $\rho_H{}^{an}(\varphi) = \rho_H(\varphi) - f_{cos}(\varphi)$.

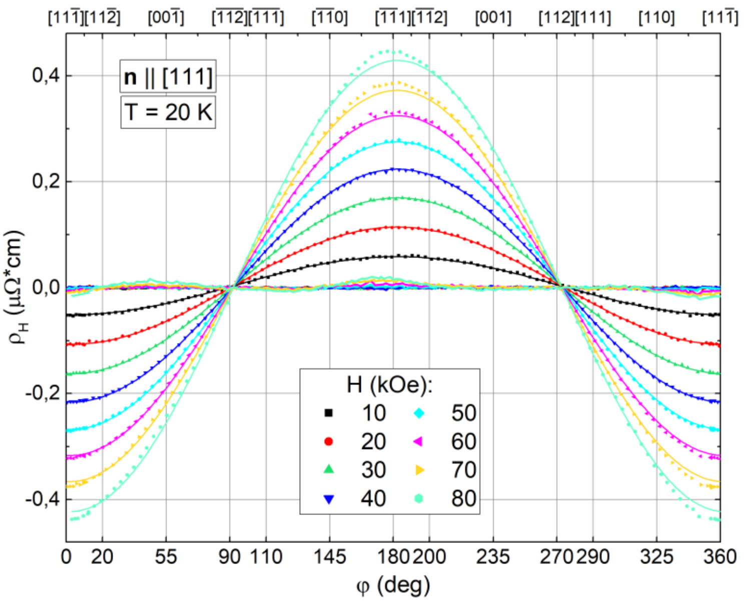

Figure S3. Approximation of the angular dependences of Hall resistivity $\rho_H(\varphi)$ at T = 20 K in magnetic fields up to 80 kOe for **n**||[111]. Symbols show the experimental values of $\rho_H(\varphi)$, thin curves present the isotropic component $f_{cos}(\varphi) \approx \rho_{H0} \cdot \cos(\varphi)$, thick curves demonstrate the anisotropic contribution $\rho_H{}^{an}(\varphi) = \rho_H(\varphi) - f_{cos}(\varphi)$.

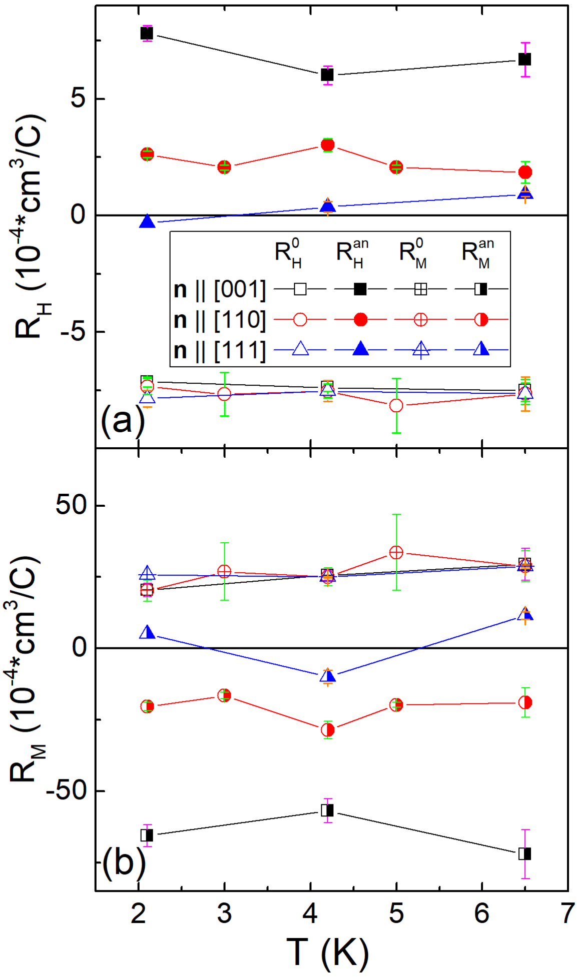

Figure S4. Coefficients (a) R_H^0, R_H^{an} of the ordinary and (b) R_M^0, R_M^{an} of the anomalous Hall effect depending on temperature for three normal vectors **n**||**H**||[001], **n**||**H**||[110] and **n**||**H**||[111].

2. Corrections of the M/B(B) data due to demagnetizing factors, to match the experimental data of ρ_H/B(B) and M/B(B).

Measurements of the field dependences of magnetization **M**(H) and of Hall resistivity ρ_H(H) were carried out on samples cut from the same single crystal, but having different geometric shapes and **H**||**n**. Therefore, for a more accurate calculation of the magnetic field induction **B** in the sample, we used a two-stage calculation with two different demagnetizing factors. The corresponding demagnetization factors for the magnetization experiments (N_1) and for the Hall resistivity experiments (N_2), respectively, are presented in Table S1.

Table S1. Values of the demagnetization factor N, depending on the type of experiment performed and the direction of the normal vector **n** to the sample surface.

	**n** || [001]	**n** || [110]	**n** || [111]
N_1, M(H) experiments	0.3972	0.3882	0.1734
N_2, ρ_H(H) experiments	0.1765	0.5324	0.54095

The upper limit of the magnetic field strength **H** in Hall resistivity ρ_H(H) measurements was 80 kOe, while when measuring the magnitude of **M**(H) it was 70 kOe in experiments with samples **n**||[001] and **n**||[110], and 50 kOe for the experiment with sample **n**||[111]. In order to match the obtained set of curves, the data of magnetization **M**(H) were linearly approximated in the range 70 - 80 kOe for all three directions of **n**.

3. Crystal orientation and quality control

3.1. X-ray powder analysis of synthesized powders and of crushed HoxLu1-xB12 single crystals revealed reflections of the UB12 structure type only, traces of RB6 or RB66 have never been detected (Fig. S5). The presence of oxygen in grown $Ho_{0.8}Lu_{0.2}B_{12}$ single crystals was checked using pulsed reductive extraction with carbon in a flow of helium gas (by gas chromatography). The oxygen content did not exceed 0.04 mass %, i.e. oxygen is chemically bound to the surface of the crushed single crystal.

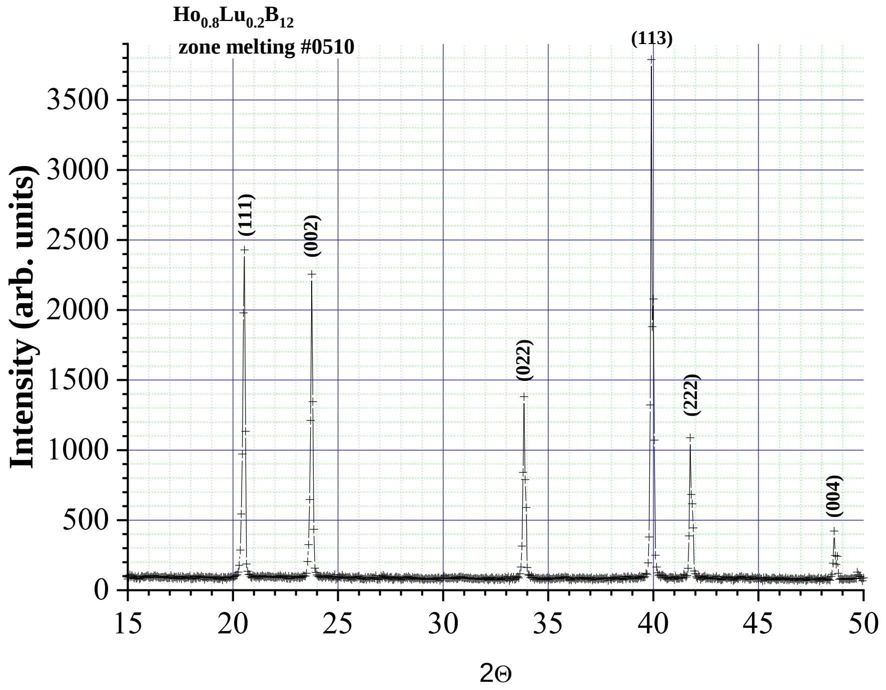

Figure S5. Typical X-ray diffraction patterns in Cu Kα radiation for crushed $Ho_{0.8}Lu_{0.2}B_{12}$ single crystals used for preparation of the experimental samples. The lattice parameter is 7.4850_3 Å. According to microprobe analysis is the real sample composition $Ho_{0.81}Lu_{0.19}B_{12}$ (see below).

3.2. All single crystals from $Ho_xLu_{1-x}B_{12}$ system were grown with oriented seeds [001], [110] and [111]. Deviations from the given orientation in as-grown crystals were eliminated during experimental sample preparation. Laue back scattering patterns from the oriented $Ho_{0.8}Lu_{0.2}B_{12}$ plates, which were used for the preparation of experimental samples, demonstrated the absence of splitting of the point reflections and confirmed the lack of domains with a misorientation more than several tenths of a degree (procedure accuracy) (see Fig. S6*a*-*c*).

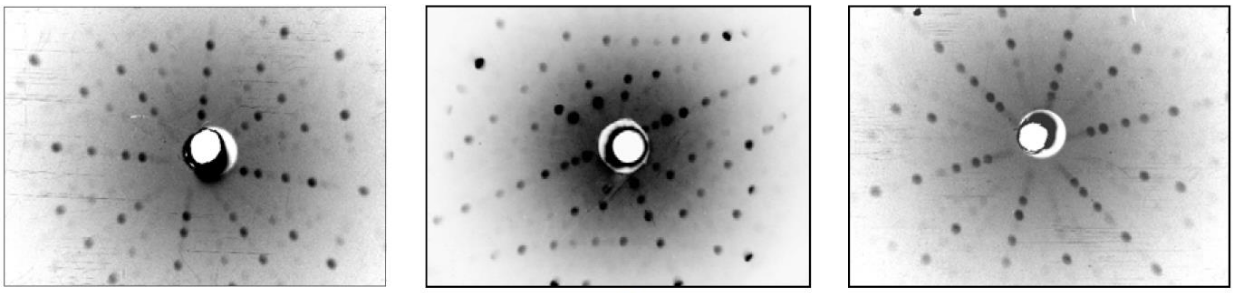

a ***b*** ***c***

Figure S6. Typical X-ray Laue backscattering patterns of the oriented plates from the same $Ho_{0.8}Lu_{0.2}B_{12}$ crystal: *a* – [100], *b* – [110], *c* – [111].

3.3. Additional verification of ~~the~~ single-domain crystals by the rocking curve control.

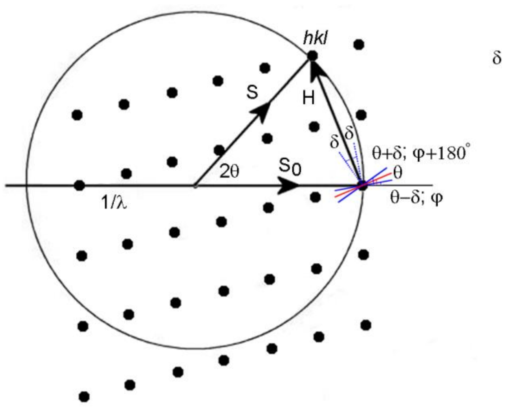

Figure S7. The idea of an experiment with a single-crystal disk in a Bruker D8 Discover x-ray diffractometer. The site *hkl* of the reciprocal lattice is located on the Ewald sphere with a radius of 1/λ. $\mathbf{S_0}$ is the incident x-ray beam; **S** is the beam diffracted at angle of 2θ to the incident one; letters θ, δ, φ denote angular rotations required to bring the site *hkl* onto the Ewald sphere. The Vector **H** = **S** - $\mathbf{S_0}$ comes from the zero site of the reciprocal lattice.

Discs about 1 mm thick and about 6 mm in diameter were cut from a single-crystal rod grown by crucible-free zone melting. The axis of the rod is oriented along the normal to one of the planes

(*hkl*) of the crystal lattice.The quality of crystals and the accuracy of coincidence of the cut plane with the (*hkl*) plane were estimated from x-ray diffraction data.The measurements were carried out in a Bruker D8 Discover diffractometer using monochromatic x-ray radiation. Fig. S7 is intended to illustrate the measurement idea. The single-crystal disk is placed at angle θ to the incident beam $\mathbf{S_0}$. The value of Bragg angle θ is obtained from the Bragg equation for the site *hkl* of the reciprocal lattice with the known period a^*_{cub}. The angle 2θ between the incident beam $\mathbf{S_0}$ and the vector **S** directed from the center of the Ewald sphere to the detector window remains unchanged during the experiment. The incident beam must be wide enough to cover the entire disc surface. The disc is mounted on a goniometric stage that can rotate around the φ axis perpendicular to the plane of the stage. The plane of the disc is adjacent to the plane of the stage. In addition, the stage with the disc can be independently rotated around the ω axis, oriented parallel to θ axis. Both of these axes are perpendicular to the plane of Fig. S7. All rotation axes of the diffractometer intersect at a point that coincides in Fig. S1 with the zero point of the reciprocal lattice.

Ideally, if the plane (*hkl*) coincides with the plane of the disk (hence, with the plane of the stage), the vector **H**(*hkl*) is directed along the φ axis, and its end lies on the Ewald sphere. In other words, the *hkl* reflex is in reflective position for any rotation of the stage around the φ axis.The plane (*hkl*) perpendicular to the plane of the figure is indicated with a red line.

In practice, the plane of the cut may be slightly different from the plane (*hkl*). As a result, the vector **H**, the end of which is displaced from the Ewald sphere, forms a small angle δ with the φ axis.The stage with the disc must be rotated around the φ axis in order to place vector **H** in the plane containing the φ axis and the incident beam $\mathbf{S_0}$. While the disk is making a full turn, this happens twice, when turning through an angle of φ and φ + 180°.In the first case, the stage with the sample must be additionally rotated by the angle $\omega_1 = -\delta$, in the second case by the angle $\omega_2 = +\delta$, in order to bring the end of vector **H** onto the Ewald sphere. These two positions of the disc and the perpendicular to its surface are indicated in Fig. S7 with blue lines. The angular positions (θ-δ, φ) and (θ+δ, φ+180°) are refined according to the intensities of diffraction peaks, combining fast φ-rotations with slow ω-rocking.

A typical rocking curve for a single-domain crystal obtained with copper radiation (λ ~ 1.54 Å) is shown in Fig. S8. The normals to the plane (*hkl*) and the plane of the crystal surface differ by angle δ = 1.2°; Two positions of the diffraction peaks (ω = θ-δ, φ) and (ω = θ+δ, φ+180°) diverge by 2δ.

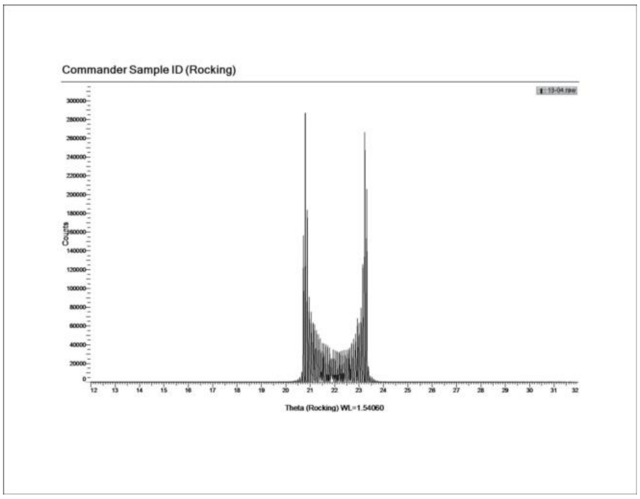

Figure S8. An example of picture from a single-domain crystal. The deviation of the crystallographic plane from the surface of the disc is 1.2°.

A more complex diffraction pattern (Fig. S9) is obtained from a crystal consisting of two blocks slightly misoriented relative to each other.The normals to the planes of the same name (*hkl*) in the first and second blocks form angles $\delta_1 = 0.8°$ и $\delta_2 = 2.4°$ with the normal to crystal surface. Judging by heights of the diffraction peaks, differently oriented blocks are present in the near-surface layer of the crystal in approximately equal amounts.

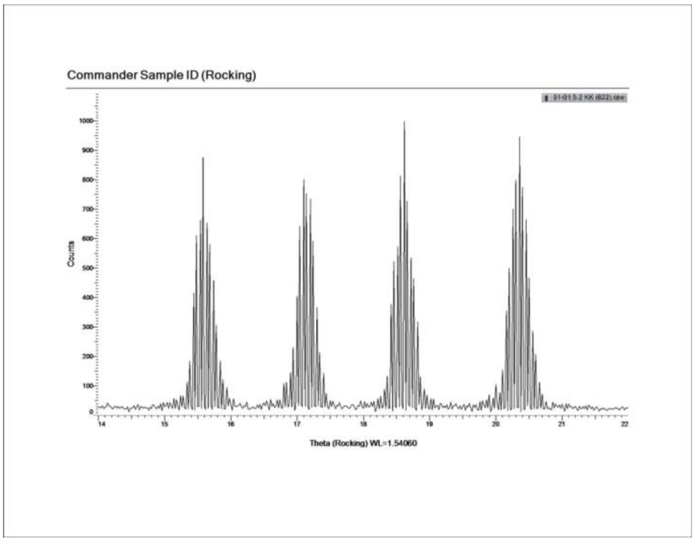

Figure S9. An example of picture from a two-domain crystal. The two blocks are represented equally in the near-surface layer. The deviations of ~~the~~ crystallographic planes of the blocks / domains from the disk surface are 0.8° and 2.4°, respectively.

3.4. The Ho/Lu ratio was estimated using a scanning electron microscope equipped with a system for energy-dispersive microprobe analysis (JEOL JXA-8200 EPMA; electron probe size 1 μ^2). Measurements were carried out at several points of the lateral cross section (R = 1, ½, 0, see Fig.10) on both sides of the single-crystalline rods in order to make sure that composition is

the same along the whole crystal. Individual binary borides (HoB_{12} and LuB_{12}) were used as reference samples. Table S2 shows the chemical composition of the $Ho_{0.8}Lu_{0.2}B_{12}$ sample.

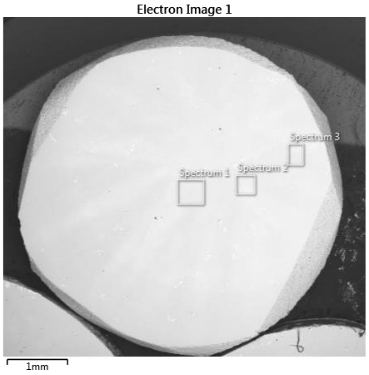

Figure S10. TEM general view of the lateral cross section of an as-grown $Ho_{0.8}Lu_{0.2}B_{12}$ single crystal (nominal).

Table S2. Results of the microprobe analysis in $Ho_{0.8}Lu_{0.2}B_{12}$ single crystal.

Point	At. %		Spectrum
	Ho	Lu	
Zone beginning			
R=0	80.7	19.3	1
R=½	81.2	18.8	2
R=1	80.9	19.1	3
Zone end			
R=0	80.5	19.5	4
R=½	81.3	18.7	5
R=1	80.8	19.2	6

Zone beginning

$Ho_{0.809\pm0.002}Lu_{0.191\pm0.002}B_{12}$

Zone end

$Ho_{0.809\pm0.004}Lu_{0.191\pm0.004}B_{12}$
